# Adult Acute Megakaryoblastic Leukemia in a Mantle Cell Lymphoma Post Treatment and Auto‐Transplant

**DOI:** 10.1002/kjm2.70013

**Published:** 2025-03-26

**Authors:** Chin‐Mu Hsu, Bi‐Hua Du, Chien Hsiao, Hui‐Hua Hsiao

**Affiliations:** ^1^ Division of Hematology‐Oncology, Department of Internal Medicine Kaohsiung Medical University Hospital Kaohsiung Taiwan; ^2^ Berkeley University Berkeley California USA; ^3^ Cancer Center Kaohsiung Medical University Hospital Kaohsiung Taiwan; ^4^ Faculty of Medicine Kaohsiung Medical University Kaohsiung Taiwan

Dear Editor,

Adult acute megakaryoblastic leukemia (AML‐M7) is a rare subtype of AML with poor outcomes and rarely be reported after therapy [1, 2]. Here we report a rare adult acute megakaryoblastic leukemia that happened 15 years after lymphoma treatment and autologous transplant.

A 61‐year‐old male patient was diagnosed with stage IV mantle cell lymphoma in October 2009. He received first‐line therapy of five courses of R‐CHOP with poor response, then salvage therapy by three courses of R‐ESHAP with partial response. He received an autologous transplant with a conditioning regimen of BEAM in September 2010. After that, he received 2 years of rituximab maintain therapy with stable disease status. Unfortunately, relapse of lymphoma was found in April 2013, and he received velcade + rituximab therapy for six courses and achieved remission after that.

However, he suffered from pancytopenia with pneumonia in December 2024. Peripheral smear showed WBC 1200/μL, Hb 8.4 mg/dL, platelet 12,000/μL, with blast cell count of 2% in the peripheral smear. Bone marrow biopsy revealed an increase of megakaryoblasts with hypolobulated dysplasia. The megakaryoblast was positive of CD34, CD117, CD61 with myelofibrosis stain of Grade 1. Cytogenetic study revealed complicated abnormalities of 49‐50, XY, +5, add(5)(q11.2), ‐7 < +8.+11,+19[11]/46, XY[14]. NGS study revealed TP53 and STAGE1 mutation. Adult AML‐M7 was diagnosed, and he received low‐dose cytarabine therapy without response and re‐induction with hypomethylation agents plus BCL‐2 inhibitors with partial response.

Adult AML‐M7 is a rare subtype of AML and accounts of only 1% in adult AML, therefore, clinical experiences and treatment options are limited [1, 2]. Most of the patients presented with cytopenia, especially thrombocytopenia. However, some of them with specific chromosome abnormalities, including the Philadelphia chromosome, chromosome 3 aberrations, del5q, del 7 [2, 3]. The outcomes are poor, with a median disease‐free survival of 6–10 months from the MDACC report [2], and the optimal treatment strategy is unclear. Our patient with cytopenia presentations and complicated chromosome/genetic abnormalities, mimics AML‐M7. Poor response to treatment implied more data for a clear conclusion on treatment and outcomes. Of interesting that the patient with a history of lymphoma post multiple therapies and auto‐transplant, makes it possible as therapy‐related myeloid neoplasms, which is still rare after such a long time from previous therapies and transplant [4, 5].

## Conflicts of Interest

The authors declare no conflicts of interest.

## Data Availability

The data that support the findings of this study are available from the corresponding author upon reasonable request.

## References

[1] L. Pagano , A. Pulsoni , M. Vignetti , et al., “Acute Megakaryoblastic Leukemia: Experience of GIMEMA Trials,” Leukemia 16, no. 9 (2002): 1622–1626. 12200673 10.1038/sj.leu.2402618

[2] Y. Oki , H. M. Kantarjian , X. Zhou , et al., “Adult Acute Megakaryocytic Leukemia: An Analysis of 37 Patients Treated at M.D. Anderson Cancer Center,” Blood 107, no. 3 (2006): 880–884. 16123215 10.1182/blood-2005-06-2450

[3] D. M. Xu , W. J. Yu , L. Xie , et al., “The Diagnostic Profile of Rare Acute Myelomegakaryoblastic Leukemia,” Leukemia & Lymphoma 62, no. 13 (2021): 3204–3211. 34477034 10.1080/10428194.2021.1955878

[4] S. Yamasaki , R. Suzuki , K. Hatano , et al., “Therapy‐Related Acute Myeloid Leukemia and Myelodysplastic Syndrome After Hematopoietic Cell Transplantation for Lymphoma,” Bone Marrow Transplantation 52, no. 7 (2017): 969–976. 28368379 10.1038/bmt.2017.52

[5] M. Akhtari , V. R. Bhatt , P. K. Tandra , et al., “Therapy‐Related Myeloid Neoplasms After Autologous Hematopoietic Stem Cell Transplantation in Lymphoma Patients,” Cancer Biology & Therapy 14, no. 12 (2013): 1077–1088. 24025414 10.4161/cbt.26342 PMC3912029

